# Ultrasonography of the recurrent laryngeal nerve in thyroid cancer: advances in clinical application and research progress

**DOI:** 10.3389/fonc.2026.1934886

**Published:** 2026-09-07

**Authors:** Binru Xia, Li Han, Ziyue Hu, Man Lu

**Affiliations:** Department of Ultrasound, Sichuan Clinical Research Center for Cancer, Sichuan Cancer Hospital & Institute, Sichuan Cancer Center, University of Electronic Science and Technology of China, Chengdu, China

**Keywords:** high-frequency ultrasound, perioperative management, recurrent laryngeal nerve, thyroid cancer, thyroidectomy, vocal cord paralysis

## Abstract

The rising incidence and detection rate of thyroid cancer have led to a concomitant increase in the number of thyroidectomy procedures performed annually. Among the postoperative complications, recurrent laryngeal nerve injury (RLNI) remains a major factor affecting patients’ quality of life. In pursuit of precision therapeutic strategies and reduced nerve injury rates,clinicians have shown growing interest in the recurrent laryngeal nerve(RLN). In recent years, high-frequency ultrasound (HFUS) has been increasingly incorporated into the perioperative management of thyroidectomy. This study is the first to address RLN management during the perioperative period for thyroid cancer. The ultrasonographic characteristics of the RLN are systematically reviewed, with particular emphasis on the clinical utility of HFUS in preoperative screening, intraoperative monitoring, and postoperative follow-up. We also discuss the inherent limitations of this technique and outline future directions. Despite these promising advances, significant challenges persist in ensuring the accuracy and standardization of ultrasonographic examinations across diverse patient populations. In conclusion, this review highlights the transformative potential of HFUS in optimizing perioperative neural monitoring and functional evaluation for thyroid cancer. However, it also notes that ultrasound cannot currently replace laryngoscopy or intraoperative nerve monitoring (IONM), though it serves as a valuable complementary tool with clear clinical utility.

## 1 Introduction

The escalating global burden of thyroid nodular disease and malignancy has precipitated a marked expansion in operative volumes (1). Among the most severe complications of these procedures, RLNI remains a major clinical concern. RLNI can be classified as temporary or permanent based on severity. Published data report an incidence of temporary RLNI ranging from approximately 0.6% to 9.6% (2), whereas permanent injury occurs at rates between 0% and 6.7% (3). Unilateral RLNI often results in vocal weakness, hoarseness, or dysphagia. However, some patients may lack typical symptoms in clinical practice, potentially increasing the likelihood of missed RLNI diagnoses. If such occult injuries remain undetected, patients having repeated surgeries are at increased risk of bilateral nerve injury (4). Despite ongoing advances in thyroidectomy and minimally invasive techniques, RLNI remains a significant concern. Therefore, preoperative identification of patients at risk for RLNI, intraoperative real-time protection, and postoperative functional monitoring have become priorities in thyroidectomy.

RLN involvement represents a critical determinant of disease staging and surgical strategy in thyroid cancer. Multiple tumor-related factors are associated with the risk of RLN invasion. For example, primary tumors in the paratracheal region or the posterior aspect of the right thyroid lobe carry an increased risk of direct RLN invasion. Therefore, active surveillance is not recommended for such lesions (5). Conversely, lesions confined to the ventral or central portion of the thyroid lobe rarely involve the nerve (6). In addition to tumor location, histological aggressiveness is also a significant risk factor for RLN invasion (7). The American Thyroid Association guidelines classify tall cell and columnar cell variants as high-risk histological subtypes warranting total thyroidectomy. This recommendation is partly attributable to the propensity of these variants to invade adjacent structures, including the RLN. Accordingly, when preoperative suspicion or confirmation of RLN involvement exists, the balance between nerve preservation and oncologic completeness must be carefully considered. Moreover, given the considerable anatomical variability and inconsistent course of the RLN, iatrogenic injury remains a significant risk during thyroid cancer resection, highlighting the importance of intraoperative real-time monitoring. Even with optimal intraoperative management, postoperative follow-up of vocal cord function is typically required to assess whether the RLN has been affected by treatment. Accordingly, comprehensive assessment of RLN integrity is imperative throughout thyroid cancer management.

At present, the conventional evaluation of the RLN primarily relies on laryngoscopy, MRI, electrophysiological testing, and IONM. However, each of these examination methods has inherent clinical limitations (**Table 1**). By comparison, ultrasonography is well suited as a first-line assessment modality for peripheral nerves because of its low-cost, real-time, dynamic assessment, and ability to continuously scan the entire course of nerves (8, 9). In recent years, advancements in ultrasound probe frequency and software algorithms have enabled clear visualization of small neural structures under HFUS. Based on its established role in other peripheral neuropathies (10, 11), accumulating evidence indicates that ultrasonography enables direct visualization of the RLN. This review aims to systematically summarize the role of HFUS in evaluating the RLN throughout the full diagnostic and treatment continuum of thyroid cancer. This narrative review is based on a literature search performed in PubMed, Embase, and Web of Science. The search primarily focused on publications from 1970 to 2026. Relevant publications were identified using combinations of keywords related to “thyroid cancer”, “thyroid surgery”, “recurrent laryngeal nerve”, “RLNI”, “perioperative management”, “high-frequency ultrasound”, “transcutaneous laryngeal ultrasonography”, “intraoperative neuromonitoring”, “IONM”, and “vocal cord paralysis”. Reference lists of selected articles were additionally screened to identify further relevant studies. Original articles, meta-analyses, systematic reviews, clinical guidelines, and historically important studies that established currently accepted concepts were included.

**Table 1:** Summary of different recurrent laryngeal nerve examination techniques, their principles, advantages, and disadvantages.

Technique	Principle	Advantages	Disadvantages
Laryngoscope	Endoscopic observation of vocal cord mobility	Evaluation of nerve function by direct vocal cord observation; Simple and convenient operation.	Poor acceptance in children and patients unable to tolerate the procedure; Lack of direct visualization of the RLN structure.
MRI	Application of high-resolution imaging based on differential water content detection via strong magnetic fields and radiofrequency waves to evaluate the RLN adjacent anatomy.	Multiplanar imaging; Radiation-free; Precise lesion localization with detailed delineation of surrounding structures.	High expense; Procedural complexity; Deficient internal neural information and the limitation in precisely visualizing long nerve segments due to resolution and field of view.
IONM	Electrophysiological assessment of neural function through stimulation and recording	Real-time intraoperative monitoring for RLN integrity assessment; Important auxiliary tools for thyroid or neck surgery.	Limited to intraoperative application; Controversial role in head and neck procedures; High expense; Limitations in feasibility and result accuracy.
Ultrasound	Imaging based on the pulse-echo principle of high-frequency sound waves	Non-invasive imaging; Real-time imaging; High resolution; Convenience and reproducibility.	High operator dependence; Dependence on patient body habitus.

RLN, recurrent laryngeal nerve.

## 2 Ultrasonographic characteristics of the RLN

**Figure 1:**
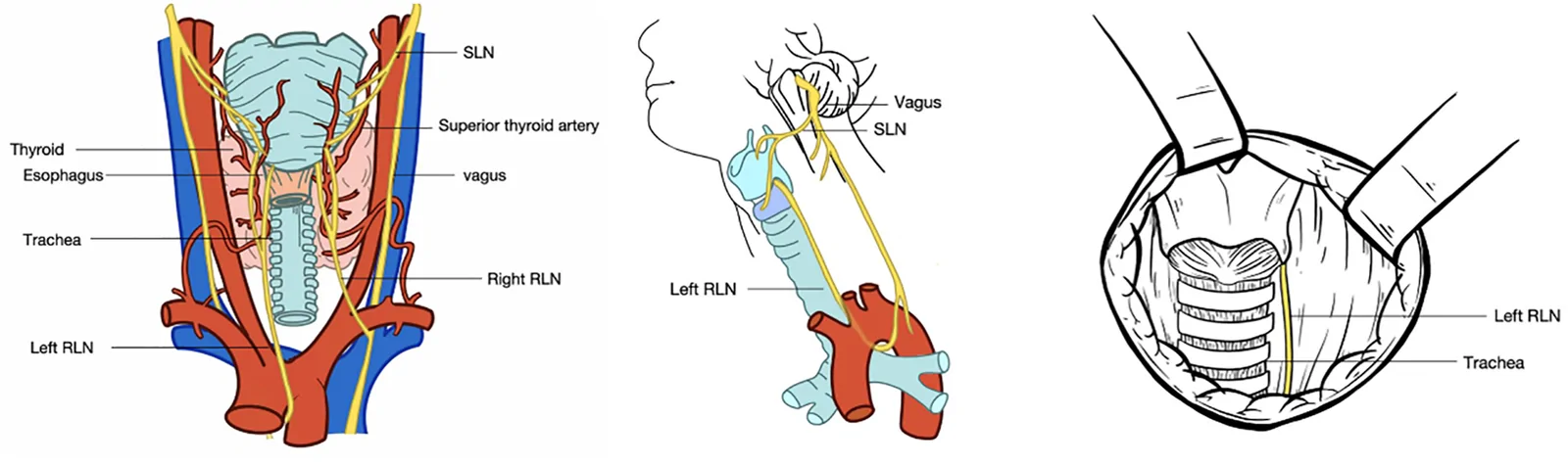
Recurrent laryngeal nerve (RLN) anatomy. Reproduced with permission (16).

**Figure 2:**
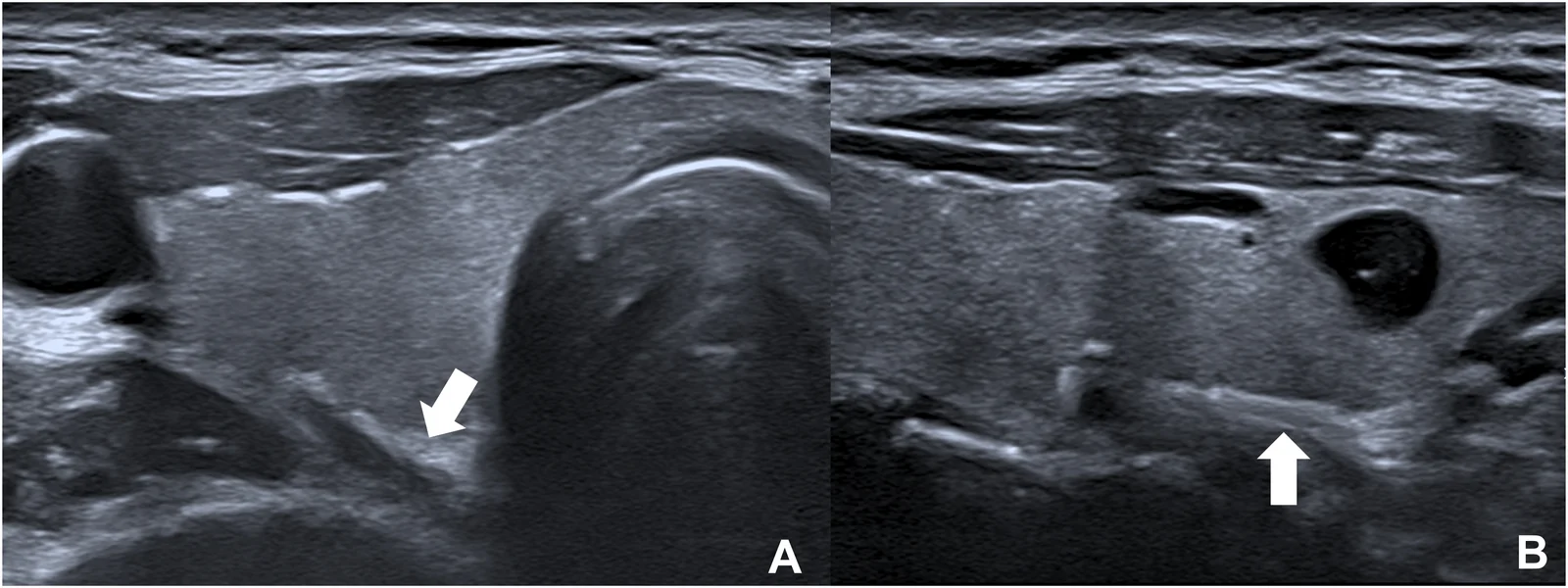
Ultrasound appearance of the right recurrent laryngeal nerve (RLN). **(A)** Transverse ultrasound image showing the RLN (white arrow). **(B)** Longitudinal ultrasound image showing the RLN (white arrow) along its course. Original images acquired at our institution.

The RLN can be evaluated on ultrasound through its spatial relationships with adjacent cervical structures, echogenicity, and surrounding landmarks (**Figure 1**). Sample et al. (12) first reported in a parathyroid ultrasound study using 5 MHz grayscale ultrasound that a linear, solid, hypoechoic structure approximately 5 mm in size could be detected within the tracheoesophageal groove (TEG), describing it as a micro neurovascular bundle. With the advancement of HFUS, previous studies have reported that the normal RLN diameter is approximately 1–2 mm, although measurements may vary according to scanning angle, transducer frequency, and individual anatomical differences (13). On transverse imaging, the RLN appears round or oval, with internal echotexture characterized by multiple hypoechoic foci distributed within a relatively homogeneous hyperechoic background. Long-axis imaging reveals multiple parallel, discontinuous hypoechoic bands separated by fine linear hyperechoic structures corresponding to the nerve fascicle sheath (14, 15) (**Figure 2**). Hu et al. (16) further validated these morphological features by employing 15–7 MHz HFUS with warm saline as an acoustic window during radical thyroidectomy. In clinical differential diagnosis, the marked echogenicity difference between the tracheal cartilage and esophageal wall can be leveraged for preliminary RLN localization via dynamic tracking of the tracheoesophageal junction. Additionally, the combined application of color Doppler further enhances differentiation of small vascular structures with similar grayscale morphology (8). This technique significantly improves the accuracy of RLN identification.

The cross-sectional area (CSA) of a nerve is considered the optimal indicator for evaluating its pathological status. To obtain the minimum CSA, the operator should place the transducer perpendicular to the nerve’s course. The inner margin tracing method is commonly recommended (17). He et al. (18) reported mean values of 4.35 ± 0.95 mm² for the left recurrent laryngeal nerve and 4.36 ± 0.95 mm² for the right recurrent laryngeal nerve (RRLN). When the nerve is too fine to accurately determine its CSA, its diameter may be used as a quantitative parameter. The normal RLN diameter is typically 1–2 mm (13). Actual measurements vary with scanning angle, probe frequency, and individual anatomical differences (19). In addition to quantitative metrics, an indistinct boundary between the epineurium and surrounding tissues may indicate nerve or adjacent pathology. Typical sonographic characteristics include increased proximal CSA, disrupted nerve bundle architecture, and hypoechoic blurring. However, the assessment of neuropathy on ultrasound images remains relatively subjective.

## 3 Role of HFUS in RLN surgical decision-making

### 3.1 Application of HFUS in RLN preoperative assessment

#### 3.1.1 Identification of anatomical variations

**Figure 3:**
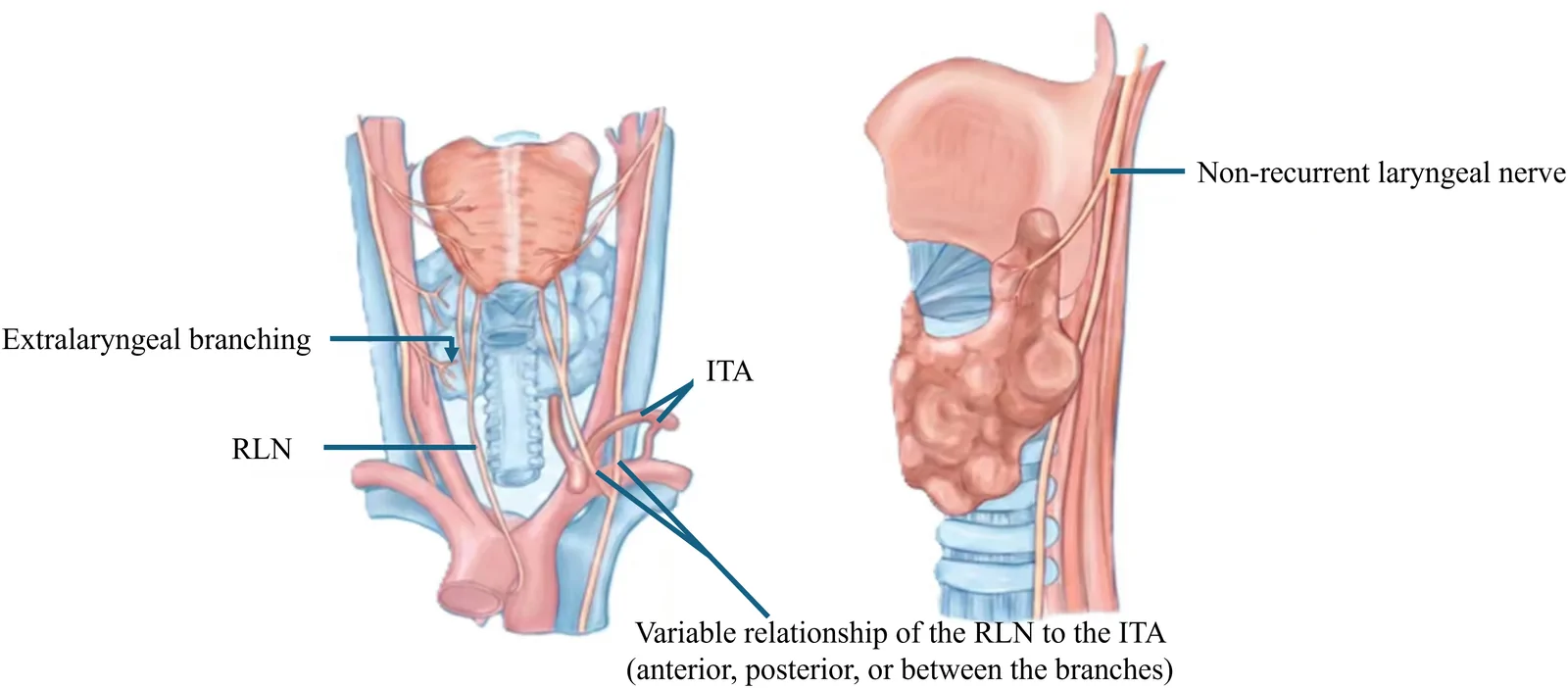
Common anatomical variations of the recurrent laryngeal nerve (RLN). ITA, inferior thyroid artery.

**Figure 4:**
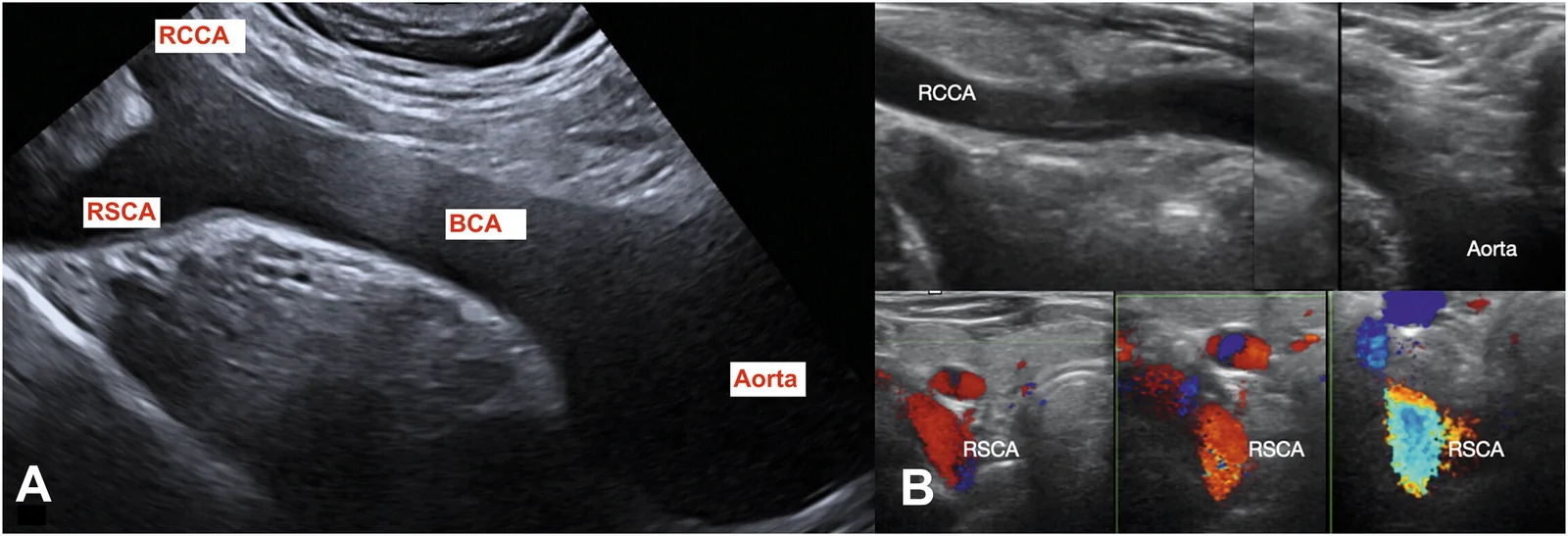
Sonographic patterns of brachiocephalic artery origin. **(A)** Normal “Y-image” bifurcation. **(B)** Anatomical variant: separate origins of the right common carotid artery (RCCA) and right subclavian artery (RSCA) from the aortic arch. Reproduced with permission (25).

Anatomical variations of the RLN are present in approximately 20–30% of individuals and may significantly affect procedural safety during surgery (14, 20). Currently, the anatomical variations of greatest clinical relevance primarily include extralaryngeal branching, the relationship between the RLN and the inferior thyroid artery (ITA), and the non-recurrent laryngeal nerve (NRLN) (**Figure 3**). However, direct visualization of extralaryngeal branches and the RLN–ITA relationship remains challenging with conventional ultrasound. In contrast, the NRLN represents the anatomical variation for which ultrasound demonstrates the highest detection sensitivity. The NRLN is a rare but clinically important variant, found almost exclusively on the right side (21). This variant is typically associated with an aberrant origin of the right subclavian artery (RSA). When the normal RSA is absent, the RRLN no longer loops around the subclavian artery and instead originates directly from the vagus nerve to enter the laryngeal cavity, thereby forming the NRLN (22). Multiple studies have consistently demonstrated that the risk of iatrogenic injury is significantly higher for the NRLN than for the conventional RLN (23, 24). Furthermore, the absence of the "Y sign" on ultrasound has been shown to reliably indicate RRLN (25, 26). This sign refers to the failure to visualize the brachiocephalic artery splitting into the right carotid and subclavian arteries, and its absence yields 99-100% sensitivity (**Figure 4**). Therefore, when NRLN is observed on ultrasound, the surgeons should be alerted to the possibility of NRLN in order to modify intraoperative anatomical strategies. However, the identification of NRLN and other RLN variants via ultrasound is highly operator-dependent and requires a thorough understanding of the embryological basis, vascular anatomy, and sonographic landmarks. False-negative results may arise if the examiner is unaware of the association between NRLN and aberrant right subclavian artery, or when the “Y sign” is incompletely evaluated due to suboptimal scanning technique or a limited field of view. Therefore, the effective use of ultrasound for detecting RLN anatomic variants necessitates not only high-resolution equipment but also dedicated training and structured interpretation protocols to minimize diagnostic errors and maximize its potential for surgical guidance.

#### 3.1.2 Assessment of the spatial relationship between the lesion and the RLN

**Figure 5:**
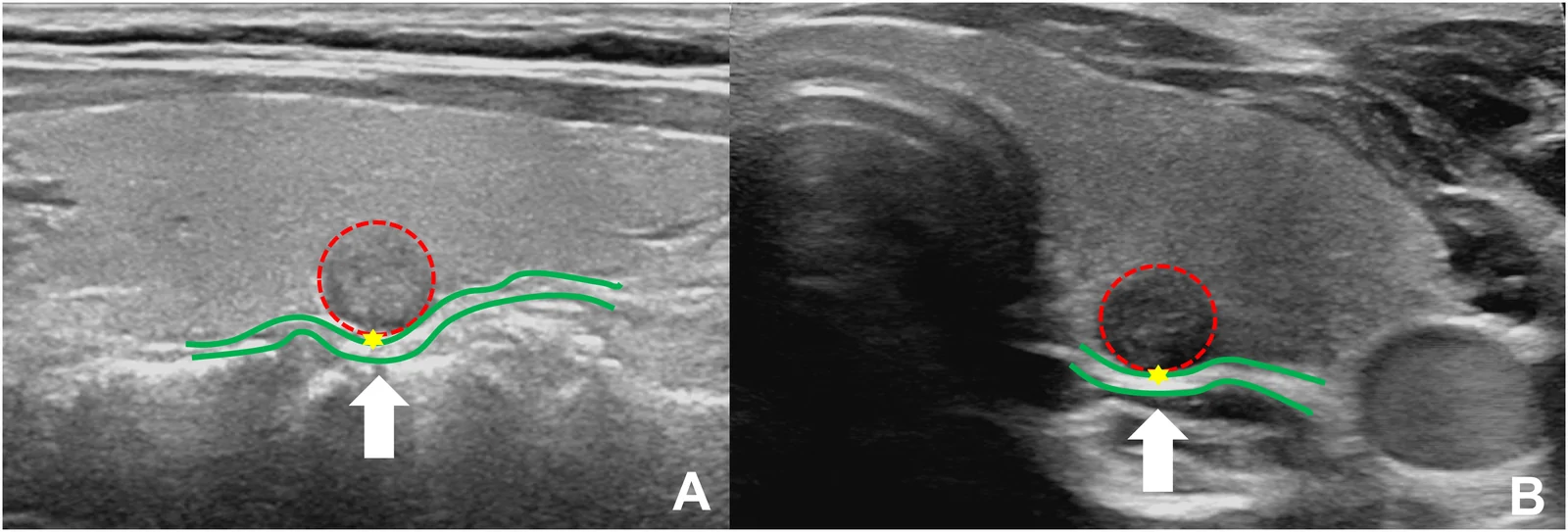
Preoperative ultrasound assessment of the spatial relationship between a thyroid nodule and the recurrent laryngeal nerve (RLN). **(A)** Longitudinal ultrasound view. **(B)** Transverse ultrasound view. In both images, the thyroid nodule is outlined by the red dashed circle, the white arrow indicates the RLN, the green lines delineate the course of the RLN, and the yellow star marks the tumor–RLN contact interface. Original images acquired at our institution.

The American Thyroid Association recommends preoperative staging and imaging for operable thyroid cancer to assess local invasion and guide surgery. Thyroid cancer frequently results in anatomical displacement of the RLN (27). Once RLN infiltration occurs, the disease is classified as stage T4a even if vocal cord mobility remains normal, rendering minimally invasive surgery contraindicated. Therefore, precise preoperative imaging provides objective evidence to evaluate options such as nerve preservation, partial resection, and RLN reconstruction (18). However, current preoperative assessment methods remain inadequate for evaluating vascular and neural involvement (28). Conventional clinical laryngoscopy has significant limitations in evaluating RLN involvement. It can only indirectly infer RLN dysfunction through abnormal vocal cord movement and cannot directly observe whether the nerve has been infiltrated by tumor. To facilitate preoperative assessment of RLN involvement, preoperative ultrasonographic evaluation is recommended (17) (**Figure 5**). Preoperative ultrasonography can be utilized to assess RLN invasion by evaluating the presence of normal thyroid parenchyma between the thyroid carcinoma and the anatomical course of the RLN. The absence of normal thyroid tissue between these structures, or protrusion of papillary thyroid carcinoma into the TEG, indicates a high risk of RLN invasion (29). Although anatomical variations may result in occult nerve invasion within apparently normal tissue in a minority of cases, TEG involvement remains a highly specific sign of invasion (30). Tumor size is positively correlated with the risk of RLN invasion. Several studies indicate that thyroid carcinomas measuring <7 mm do not exhibit RLN invasion, regardless of risk stratification. However, tumors ≥7 mm warrant careful evaluation, as the risk of RLN invasion is substantially higher (31, 32). Additionally, morphological alterations of the nerve observed on ultrasound enable preliminary assessment of invasion. Previous studies have reported that sonographic features suggestive of RLN invasion include loss of the normal fascicular echotexture along the longitudinal axis, a tumor-to-RLN distance of <1 mm, and an indistinct boundary between the RLN and the tumor (18, 33). Hu et al. further refined this morphological feature, noting that the invaded segment of the RLN is narrower, whereas the non-invaded portion appears thicker due to edema (16). This finding provides additional quantitative evidence for preoperative assessment of nerve invasion. As a noninvasive modality, preoperative ultrasonography can establish a baseline for preoperative vocal cord mobility, serving as a reference for perioperative functional comparison.

#### 3.1.3 Assessment of vocal cord mobility by transcutaneous laryngeal ultrasonography

Laryngoscopy remains the primary technique for assessing preoperative RLN function (34). But laryngoscopy may also overlook early RLN involvement before obvious abnormalities in vocal cord mobility appear (5). Therefore, transcutaneous laryngeal ultrasonography (TLUSG) represents a promising method for assessing RLN function in the perioperative setting. TLUSG uses the cricoid cartilage as an acoustic window to indirectly assess RLN functional integrity through dynamic observation of vocal cord adduction and abduction. Woo et al. (35) and Knyazeva et al. (36) further proposed using gel pads or water-filled gloves between the probe and skin to improve acoustic window conditions. Current evidence strongly supports the use of TLUSG for initial screening of RLN function and recommends its incorporation into routine preoperative neck ultrasound examination (37, 38). Gambardella et al. (39) conducted a multicenter prospective study comparing preoperative TLUSG with laryngoscopy. The two modalities demonstrated excellent concordance. TLUSG identified vocal cord dysfunction with sensitivity and specificity of 96.8% and 95.6% respectively. Similarly, Carniero-Pla et al. (40) also reported 100% sensitivity and 98% specificity. However, a large retrospective study by Knyazeva et al. (41) (n=668) reported comparable specificity but a lower sensitivity of 66.7%. These discrepancies may be related to operator experience, equipment resolution, and differences in the anatomical characteristics of the included population. In addition, the accuracy of TLUSG remains constrained by multiple factors. Age-related calcification and cartilage angulation may exacerbate acoustic attenuation, resulting in degraded image quality and impaired vocal fold identification. Additionally, postoperative hematoma, inflammation, and severe infection can also interfere with the examination. It is also important to recognize that TLUSG evaluates vocal fold mobility as an indirect indicator of RLN function, rather than directly assessing nerve integrity itself, which represents a limitation compared with IONM. Overall, TLUSG demonstrated excellent specificity. Nevertheless, given the potential variability in sensitivity, laryngoscopy is still recommended for negative cases with poor sonographic visualization or equivocal findings.

### 3.2 Application of HFUS in RLN intraoperative monitoring

In thyroidectomy or parathyroidectomy, RLNI with an intact gross appearance is more common than nerve transection (42). Wagner et al. (43) reported that in total lobectomy, the rate of permanent paralysis in cases without RLN identification increased significantly from 3.8% to 7%. Kale et al. (44) further demonstrated that intraoperative identification of variations and complete dissection of the RLN and its surrounding branches significantly reduced the incidence of permanent postoperative paralysis. Therefore, intraoperative anatomical identification of the RLN has been increasingly advocated as a surgical strategy to reduce injury. Nevertheless, given the limitations of macroscopic assessment, functional verification using imaging or electrophysiological techniques remains an essential supplement.

IONM is routinely employed in thyroidectomy to assist surgeons and reduce vocal cord paralysis (VCP) (45). Meta-analyses by Zheng (46) and Merchavy (47) both demonstrated that IONM can lower the incidence of transient and permanent RLNI. However, the implementation of IONM often relies on expensive disposable electromyography monitoring tracheal tubes. In this context, ultrasonography is emerging as a promising tool for real-time intraoperative RLN visualization and functional assessment.

**Figure 6:**
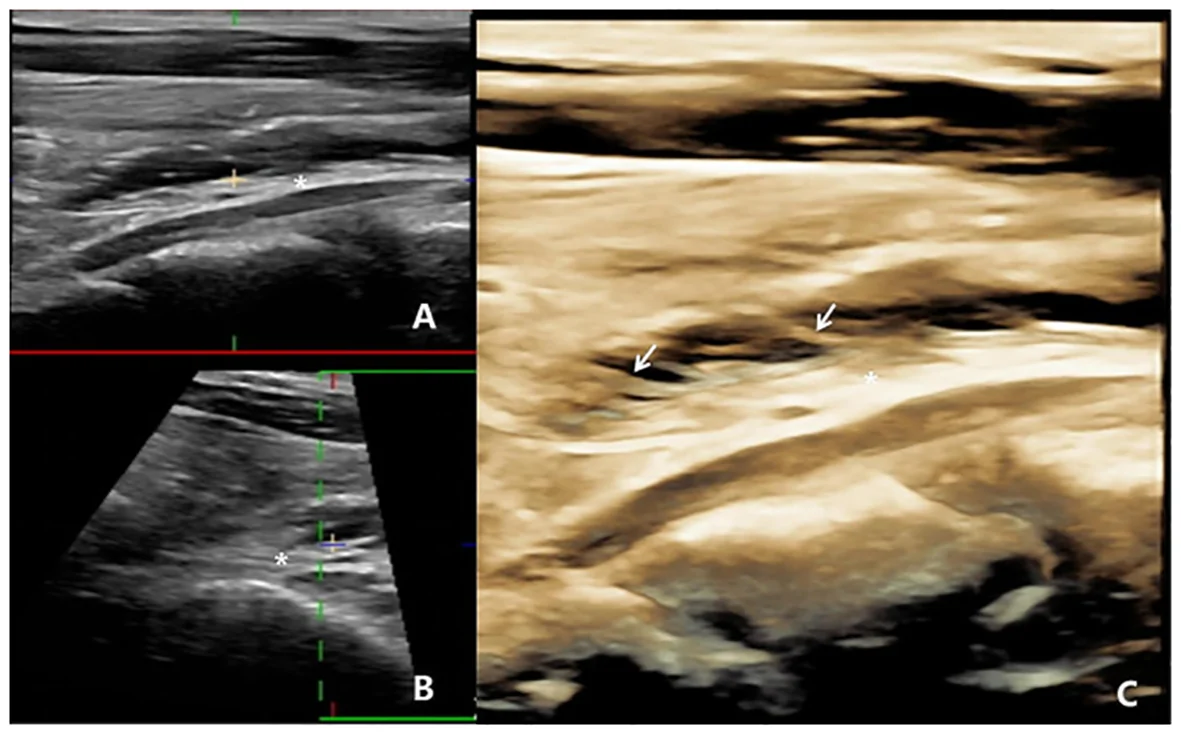
3D imaging of the recurrent laryngeal nerve (RLN) after water isolation. **(A)** Longitudinal view of the RLN (asterisk). **(B)** Transverse view of the RLN (asterisk). **(C)** 3D ultrasound imaging showing multiple small branches of the RLN (asterisk) (white arrows). Reproduced with permission (51).

In surgeries with limited visual exposure, intraoperative ultrasound examination is essential for assessing the relationship between the RLN and surrounding structures. Gürsoy et al. (48) and Haldeman et al. (49) have confirmed that intraoperative ultrasound complements traditional localization techniques. It enables dynamic visualization of cervical nerves relative to surrounding muscles and vessels, eliminating blind exploration. Willsey et al. (50) further proposed that sterile saline injection creates a protective buffer by expanding the nerve-tissue interval. In our previous work, we demonstrated that intraoperative fluid injection with normal saline between the thyroid capsule and the RLN creates a protective fluid plane and improves sonographic delineation of the RLN, thereby facilitating real-time guidance for dynamic nerve avoidance during ablation (51) (**Figure 6**). In addition, intraoperative TLUSG demonstrates 98.1% accuracy in verifying RLN functional integrity (52). Fung et al. (53) reported that TLUSG exhibits comparable detection accuracy to electromyography during IONM. Accordingly, they advocated for the routine use of TLUSG as a monitoring tool during thyroid radiofrequency ablation procedures. This technique generalizes easily and has a short learning curve. It requires approximately 40 procedures to achieve consistent and reliable assessment (54).

Intraoperative ultrasonography effectively visualizes the RLN and its anatomical relationships with adjacent vessels and lesions. However, the diagnostic performance of ultrasound is influenced by both operator expertise and imaging conditions, including transducer frequency selection and intraoperative acoustic window quality. Furthermore, the current literature lacks sufficiently prospective studies to confirm that ultrasound alone can reduce RLNI rates or replace IONM. Therefore, ultrasound should be regarded as a complementary imaging method to IONM rather than a substitute. Its primary value lies in providing additional anatomical guidance, particularly in selected high-risk scenarios where distorted anatomy or prior surgery complicates RLN identification.

### 3.3 Application of HFUS in RLN postoperative follow-up

**Figure 7:**
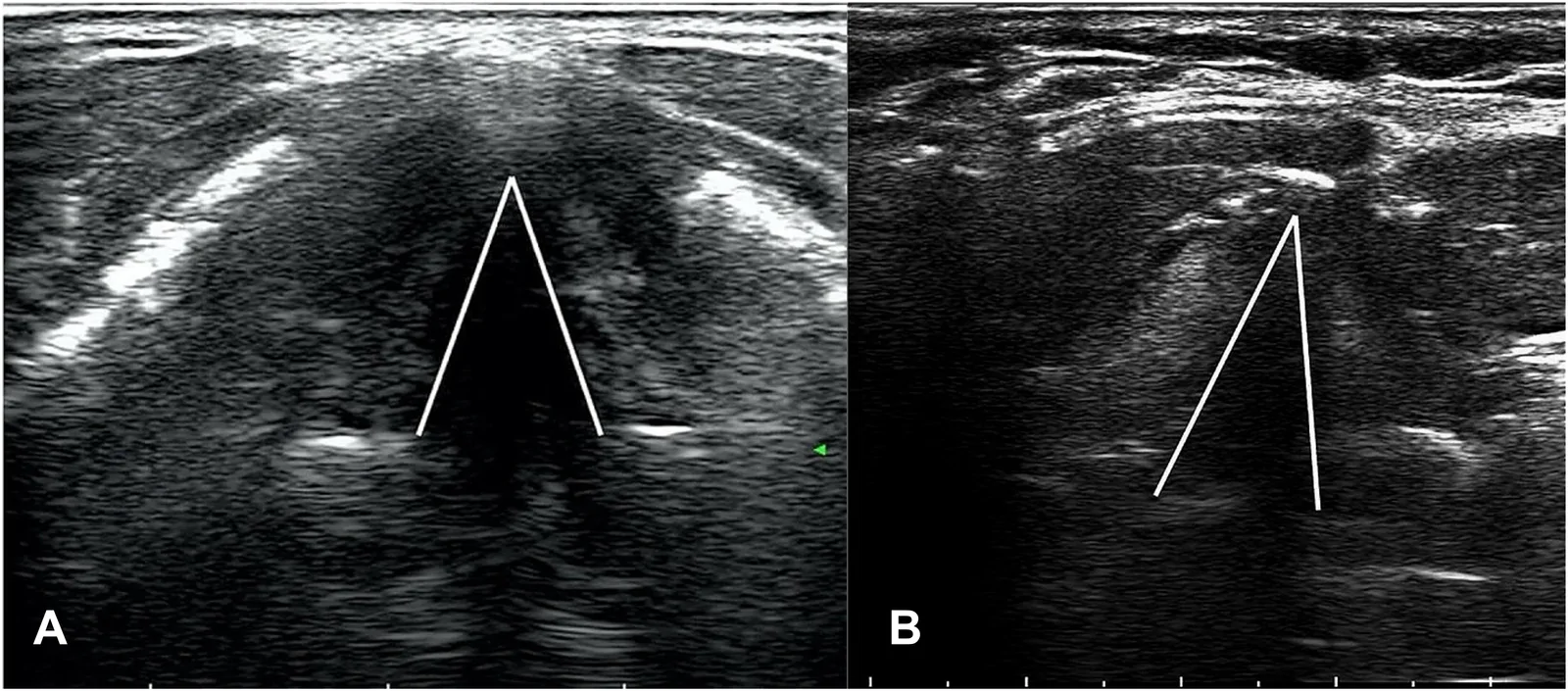
Postoperative normal versus abnormal vocal cord mobility. **(A)** Bilateral normal vocal cords abduction. **(B)** Left vocal fold movement impairment. Reproduced with permission (64).

The primary objective of assessing RLN function after surgery is the early detection and diagnosis of VCP. Ultrasonography allows for rapid assessment of vocal cord mobility and the evaluation of morphological changes in nerves and muscles. Several studies have reported that TLUSG enables early identification of RLN and superior laryngeal nerve palsy following thyroidectomy (55). However, the postoperative use of TLUSG alone is inconsistently supported by existing evidence, with reported sensitivities for VCP ranging from 33% to 100% (56). Singh et al. (57) found that the diagnostic accuracy of TLUSG slightly decreased postoperatively compared to preoperatively, though it remains a useful supplement to conventional laryngoscopy techniques. Gao et al. (58) further proposed that TLUSG may be an effective alternative to laryngoscopy for evaluating patients with postoperative voice dysfunction. In contrast, some studies challenge the routine use of postoperative TLUSG for functional evaluation. Sidhu et al. (59) concluded that TLUSG cannot reliably evaluate vocal cord function after surgery. Kandil et al. (60) also observed that postoperative TLUSG of the vocal cords presents greater challenges than preoperative evaluation. Borel et al. (61) further confirmed this limitation through a prospective study. Although the sensitivity of TLUSG remains controversial, the majority of studies have demonstrated a favorable negative predictive value (39, 62, 63). Given the overall low incidence of postoperative VCP, normal vocal fold mobility on TLUSG strongly predicts intact RLN function. Therefore, TLUSG can serve as a first-line screening tool for initial evaluation in patients with normal postoperative phonation and normal vocal fold mobility. When sonographic visualization is inadequate or abnormal findings are detected, laryngoscopy remains the gold standard for definitive diagnosis (64) (**Figure 7**).

## 4 Discussion

HFUS has many characteristics that render it a valuable modality for both the visualization and functional assessment of the RLN. In recent years, perioperative assessment and preservation of the RLN have evolved toward a framework integrating multimodal fusion, multidimensional imaging, and intelligent assistance. Future research should aim to overcome the limitations of a single monitoring modality by integrating HFUS, which not only facilitates neural localization but also provides information on the type and severity of neuropathy (65). The combined intraoperative use of HFUS and electrophysiological signals from IONM enables simultaneous assessment of both the morphology and function of the RLN. In recent years, there has been a pervasive utilization of three-dimensional ultrasonography imaging technology, offering the potential to overcome the limitations of two-dimensional imaging by providing volumetric data that enable precise morphological and functional assessment. Currently, three-dimensional ultrasonography of peripheral nerves such as the sciatic nerve and brachial plexus has been extensively reported (66–68). However, its application to the RLN remains relatively limited. Three-dimensional ultrasonography enables the visualization of spatial relationships between the RLN and adjacent structures, including the thyroid, trachea, and vasculature. It is uniquely valuable in delineating neural courses and identifying anatomical variants, particularly when applied to cases with complex anatomy or high-risk features. Elastography, by quantitatively assessing alterations in nerve stiffness from a biomechanical perspective, addresses the unmet need for tissue characterization. The application of elastography in the neuromuscular and musculoskeletal domain has progressively matured, with carpal tunnel syndrome representing the most extensively investigated condition (69–71). Relevant reports have also been documented in conditions such as ulnar neuropathy, brachial plexopathy, and various peripheral polyneuropathies (72–75). Although elastography studies specifically targeting the RLN remain relatively scarce, the aforementioned findings from peripheral nerve research provide a robust theoretical foundation for RLN assessment. Computer-based artificial intelligence is currently a major research focus (76). In the future, it is expected to enable automated analysis and diagnosis of neuropathies, as well as to provide quantitative assessments for monitoring treatment efficacy (77). This would enhance diagnostic accuracy while reducing dependence on operator expertise and minimizing inter-operator variability (78).

Despite the promising potential of ultrasound for RLN assessment, several important limitations must be acknowledged. First, operator dependency and lack of standardization remain pervasive across all clinical scenarios. Second, standardized reference ranges for quantitative metrics have not been established, and the available evidence on HFUS performance is derived predominantly from relatively small single-center cohorts. Furthermore, no large-scale multicenter study has been conducted to establish population-validated normative values. Consequently, the utility of existing data for individual clinical decision-making remains limited. Third, the precise clinical role of ultrasound in RLN assessment remains to be defined. While TLUSG shows promise as a first-line screening tool in postoperative patients, current evidence does not support replacing laryngoscopy with ultrasound for standalone vocal cord assessment. Similarly, it does not support substituting IONM with ultrasound for the prevention of RLNI. On one hand, the nerve is characterized by its slender caliber, deep anatomical location, and considerable anatomical variability. Consequently, adequate visualization remains challenging or even unattainable in patients with obesity, thyroid cartilage calcification, goiter, or prior neck surgery. On the other hand, large-scale prospective controlled trials are lacking to clarify whether ultrasound provides additional clinical value beyond visual nerve identification and IONM. Given the current evidence, large-scale, multicenter prospective studies with standardized imaging protocols are urgently needed to validate the clinical utility of HFUS and define its precise role in routine practice. Future research should also prioritize the establishment of a standardized perioperative protocol for RLN visualization and protection, and further explore the potential of emerging technologies to optimize patient outcomes and quality of life.

**Figure 8:**
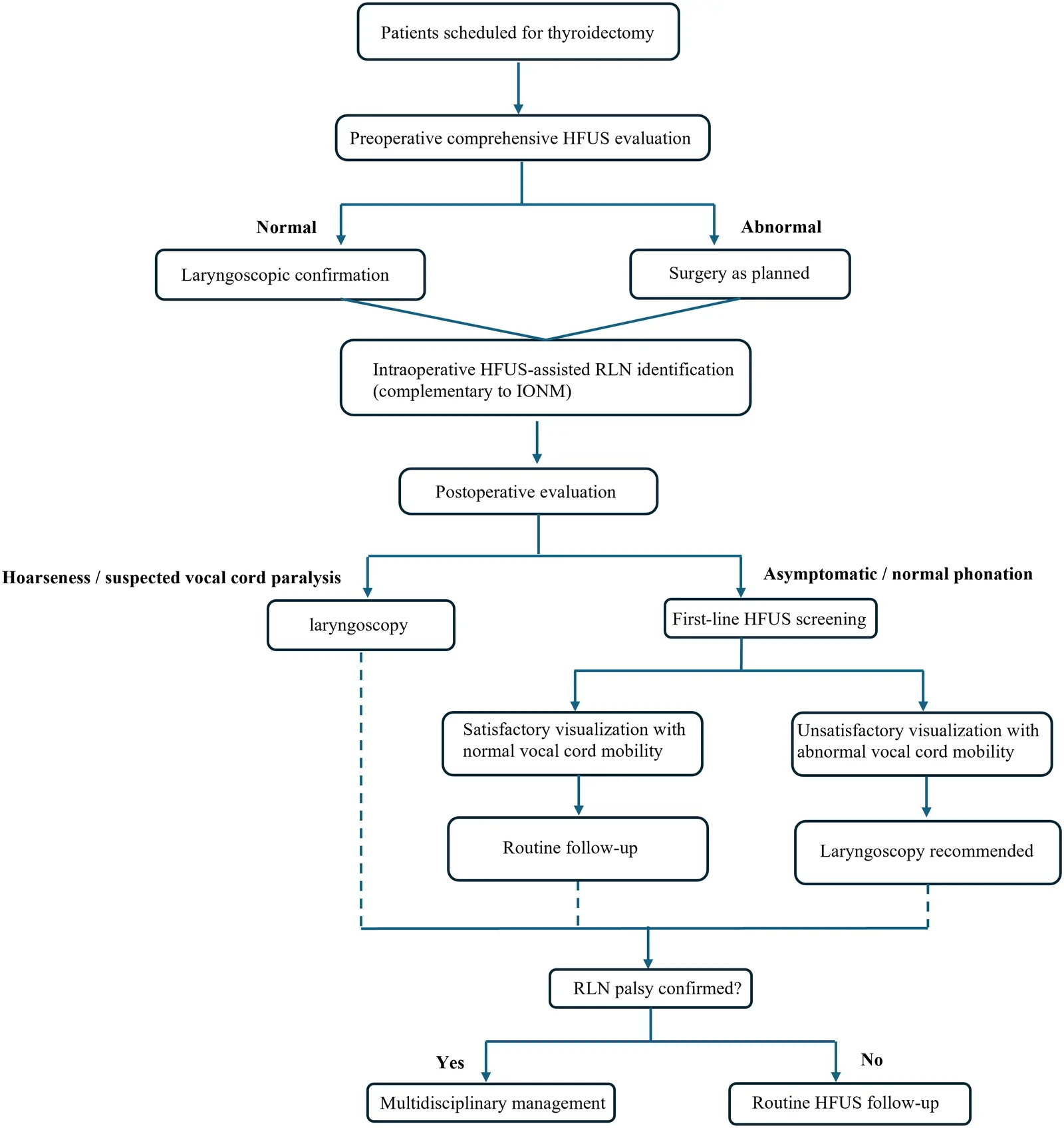
Proposed clinical algorithm for high-frequency ultrasound integration into perioperative recurrent laryngeal nerve assessment.

To provide a practical overview of these clinical scenarios and a structured decision-making framework, we summarize the proposed applications of HFUS during the perioperative period in **Table 2** and present a corresponding clinical algorithm in **Figure 8**.

**Table 2:** Clinical applications of high-frequency ultrasound in the perioperative period for thyroid cancer.

Phase	Clinical context	Role of HFUS	Key considerations
Preoperative	Preoperative evaluation of patients scheduled for thyroidectomy	Identification of RLN anatomical variants (particularly the non-recurrent laryngeal nerve); Assessment of spatial relationship between RLN and thyroid lesions (tumor invasion/adhesion); Evaluation of baseline bilateral vocal fold morphology and mobility.	Indicated for patients scheduled for thyroidectomy, particularly those at higher risk of RLN injury; Positive findings on vocal fold mobility assessment require laryngoscopic confirmation; Identification of anatomical variants and spatial relationships is operator-dependent.
Intraoperative	Real-time RLN localization and functional assessment	Adjunctive imaging tool for anatomical identification of the RLN; complementary to IONM.	Cannot replace IONM; Limited by sterile field, probe accessibility, and surgical manipulation; Remains an exploratory tool.
Postoperative (Early)	Screening for vocal fold mobility in patients with normal phonation	First-line screening modality to rule out vocal fold palsy in asymptomatic patients with normal voice.	Laryngoscopy is not required when visualization is adequate and fold mobility normal; Laryngoscopy remains the gold standard when visualization is inadequate or abnormal findings are detected.
Postoperative (Follow-up)	Serial monitoring of vocal fold function recovery	Dynamic assessment of mobility recovery; Potential integration into routine thyroid ultrasound examination.	Optimal timing and frequency of follow-up remain unstandardized; Recommended as a complement to routine sonographic surveillance.

RLN, recurrent laryngeal nerve; IONM, intraoperative neuromonitoring; HFUS, high-frequency ultrasound.

HFUS is a rapidly growing technology with substantial potential for expanding its clinical utility in the future. Although the advancement of HFUS diagnostic tools remains fraught with challenges, it is playing an increasingly pivotal role in the visualization and functional assessment of the RLN. Its dynamic scanning capability, coupled with real-time symptom correlation, offers distinct advantages, frequently revealing neuropathies that static imaging modalities may fail to detect. With continued technological refinement and the accumulation of clinical experience, HFUS is expected to assume an increasingly prominent role in the perioperative management of the RLN, ultimately contributing to the reduction of iatrogenic injury and the improvement of patient outcomes.
